# A New Framework to Implement Model-Informed Dosing in Clinical Guidelines: Piperacillin and Amikacin as Proof of Concept

**DOI:** 10.3389/fphar.2020.592204

**Published:** 2020-12-16

**Authors:** Stan J. F. Hartman, Joost G. E. Swaving, Stijn W. van Beek, Bianca D. van Groen, Marika de Hoop, Tjitske M. van der Zanden, Rob ter Heine, Saskia N. de Wildt

**Affiliations:** ^1^ Department of Pharmacology and Toxicology, Radboud Institute of Health Sciences, Radboudumc, Nijmegen, Netherlands; ^2^ Department of Pharmacy, Radboud Institute of Health Sciences, Radboudumc, Nijmegen, Netherlands; ^3^ Intensive Care and Department of Pediatric Surgery, Erasmus MC-Sophia Children’s Hospital, University Medical Center Rotterdam, Rotterdam, Netherlands; ^4^ Dutch Knowledge Center Pharmacotherapy for Children, Den Haag, Netherlands; ^5^ Royal Dutch Pharmacist Association (KNMP), The Hague, Netherlands; ^6^ Department of Intensive Care Medicine, Radboud Institute of Health Sciences, Radboudumc, Nijmegen, Netherlands

**Keywords:** pharmacokinetics, model-informed dosing, clinical implementation, critically ill children, piperacillin, amikacin

## Abstract

**Background**: Modeling and simulation is increasingly used to study pediatric pharmacokinetics, but clinical implementation of age-appropriate doses lags behind. Therefore, we aimed to develop model-informed doses using published pharmacokinetic data and a decision framework to adjust dosing guidelines based on these doses, using piperacillin and amikacin in critically ill children as proof of concept.

**Methods**: Piperacillin and amikacin pharmacokinetic models in critically ill children were extracted from literature. Concentration-time profiles were simulated for various dosing regimens for a virtual PICU patient dataset, including the current DPF dose and doses proposed in the studied publications. Probability of target attainment (PTA) was compared between the different dosing regimens. Next, updated dosing recommendations for the DPF were proposed, and evaluated using a new framework based on PK study quality and benefit-risk analysis of clinical implementation.

**Results**: Three studies for piperacillin (critically ill children) and one for amikacin (critically ill pediatric burn patients) were included. Simulated concentration-time profiles were performed for a virtual dataset of 307 critically ill pediatric patients, age range 0.1–17.9 y. PTA for unbound piperacillin trough concentrations >16 mg/L was >90% only for continuous infusion regimens of 400 mg/kg/day vs. 9.7% for the current DPF dose (80 mg/kg/6 h, 30 min infusion). Amikacin PTA was >90% with 20 mg/kg/d, higher than the PTA of the DPF dose of 15 mg/kg/d (63.5%). Using our new decision framework, altered DPF doses were proposed for piperacillin (better PTA with loading dose plus continuous infusion), but not for amikacin (studied and target population were not comparable and risk for toxicity with higher dose).

**Conclusions**: We show the feasibility to develop model-informed dosing guidelines for clinical implementation using existing pharmacokinetic data. This approach could complement literature and consensus-based dosing guidelines for off-label drugs in the absence of stronger evidence to support pediatricians in daily practice.

## Introduction

The Dutch Pediatric Formulary (DPF) provides pediatric dosing recommendations for all drugs used in children the Netherlands (van der Zanden et al., 2017). This includes drugs used off-label by indication and/or age group, but also drugs approved for use in children. If emerging evidence suggests the labelled dose to be suboptimal, the DPF adjusts the dose to reflect up-to-date evidence. These best-evidence doses are based on a standardized benefit-risk analyses using literature data, including doses used in clinical trials and expert opinion. The DPF overcomes the current information gap for physicians when a medical need to prescribe a drug to children is evident and the registered pediatric dose is lacking or believed suboptimal due to emerging new data.

Drug disposition rapidly changes during growth and development, due to maturation of the processes involved in absorption, distribution, metabolism and excretion (Kearns et al., 2003). Not addressing these differences between adults and children might cause suboptimal exposure, lack of efficacy or adverse effects in children (Kearns, 2010; Tuleu and Breitkreutz, 2013). Pediatric pharmacokinetic (PK) data, reflecting these age-related changes can be used to simulate dosing regimens reaching therapeutic and safe exposures. Indeed, model-informed dosing is increasingly used to support dosing recommendations, but implementation in clinical care of such dosing guidelines is lagging behind (Darwich et al., 2017; Keizer et al., 2018). Moreover, many pediatric PK publications do not include dosing simulations and/or proposals for dosing.

We hypothesized that existing, published PK data can also be used to generate dosing recommendations and be used to optimize existing dosing recommendations for children, to be implemented in clinical dosing guidelines, such as the DPF. The aim of our study was to develop a framework using model-informed doses based on published PK studies, as a complementary tool to generate model-informed, evidence-based dosing guidelines.

## Materials and Methods

### Literature and Selection of Drugs

As proof of concept, we focused on the dosing regimens of antibiotics in critically ill children, as concentration targets are available, to enable concentration-based simulations (Tsai et al., 2015). Moreover, these drugs are relatively well studied in children with published pharmacokinetic data ranging from well-validated population PK (pop-PK) studies including dose simulations, to more basic studies simply reporting drug concentrations (Hartman et al., 2019).

We selected publications using pop-PK modelling as these models include interindividual variation (IIV) as a parameter. This provided the possibility to study the full target range and identify the risk of over- or underdosing with the simulated doses. We extracted any information on model structure of the final model, differential equations, covariate relationships, PK-parameter estimates (volume of distribution (Vd) and clearance (Cl)), IIV, and residual error model. Additionally, we evaluated concentration-time profiles in the publications for peak (Cmax) and trough (Cmin) concentrations in order to compare our results to the published studies. Lastly, we identified whether the publication provided a dose advice for critically ill children.

This information was used to select suitable drug candidates aiming to study one drug with and one drug without dosing simulations and recommendations in the manuscript. Both drugs had to have dosing recommendations in the DPF.

### Generating Dosing Regimens

PK models were implemented in R and R-studio using the published PK parameters (R version 3.6.2, R-studio version 1.2.1335, R Core Team 2013) with additional package “mrgsolve” and evaluated using “ggplot2” (ggplot2, 2016; Mrgsolve, 2020). Model codes were requested from the authors of the included publication. We only received the model code from De Cock et al. which was used to verify our version of the model (De Cock et al., 2017). The models were written in line with the “mrgsolve user guide.” Development and evaluation of the rebuilt models was performed in three steps (detailed in *Step 1: Implementation of Models in a Standardized R-Script*, *Step 2: Extrapolation; Generating a Dosing Advice*, and *Step 3: Decision Framework for Best Evidence Dosing Guidelines*):

#### Step 1: Implementation of Models in a Standardized R-Script

The first step consisted of rebuilding the model as described in the original article for a specific antibiotic. This step was performed to check validity of the models, as rebuilding the original models should provide similar concentration-time outcomes to the published article. We used the dosing advices in the original articles as input for the model.

R-scripts of the models were written in a fixed format including covariate relationships, PK-parameter values, population characteristics and specific dosing regimens (as described above). Other model-specific characteristics such as model structure, differential equations and error-models were dependent on the number of compartments and the type of error model presented in the article.

The R-scripts were checked for purposes of quality control by two experienced pharmacokinetic modelers (RTH and SVB). This ensured the models in the script were correctly adapted and represented the models of the original articles.

#### Step 2: Extrapolation; Generating a Dosing Advice

After evaluating the implementation of the model in step 1, a dosing advice was generated based on simulated concentration-time profiles, toxicity thresholds and the PK targets of efficacy and/or safety.

### Simulation Patient Population

For our simulations we used a virtual PICU patient dataset with anonymous demographic and relevant covariate data of critically ill children, 1 m–18 y of age, admitted to the PICU of the Radboudumc in 2018. This was done to ensure we had a virtual patient population that closely mimics the target population for the new dosing regimen. Data were obtained from the electronic patient records and included weight, height, postmenstrual age, postnatal age, gender and eGFR calculated with the creatinine-based revised Schwartz formula (Schwartz et al., 2009). Patients were excluded if more than two of the requested demographic characteristics were missing.

Based on the Dutch Law on Human Drug Research, formal ethical approval by an institutional review board or informed consent were not needed as anonymized clinical patient data were used.

### Concentration (PK) Targets

Different PK targets for the efficacy and safety of antibiotics are used, dependent on the properties of the drug. Based on the drugs chosen for the simulation we identified the optimal PK targets. We used epidemiological cut-off values of minimal inhibitory concentrations (MIC) as target values aiming for a clinically relevant, worst-case scenario. For this we used the epidemiological cut-off for MIC values provided by the European Committee on Antimicrobial Susceptibility Testing (EUCAST) for each drug. The relevance of protein binding was evaluated and obtained from literature data of comparable patient populations.

### Dose Simulations

Using 100 iterations of the PICU simulation dataset, concentration-time curves were created and compared to Cmax, Cmin and IIV presented in the original articles. Subsequently, simulations of concentration-time profiles were performed for different dosing regimens.

First, the current dose in the Dutch Pediatric Formulary (DPF) was simulated (Dutch Pediatric Formulary-Amikacin; Dutch Pediatric Formulary-Piperacillin). For the drug with dose advice in the paper(s) we also used the advised dose as input for these simulations. For the drug without a dose advice in the paper, several dosing regimens were examined for reaching the determined PK-targets, using a “trial and error”-principle with predefined dose increments, based on the drug’s DPF dose and dose intervals of at least 12 h. We compared the simulation results between the DPF dose and these “trial and error” doses.

Cross-checks of these doses were performed between the different models for additional insight in applicability and robustness. Additionally, probability of target attainment (PTA) was determined by the proportion of patients that reach selected target concentrations for safety and/or efficacy. PTA was determined for current and selected optimal dosing regimens, in order to quantify the improvements in PTA of a new dosing regimen.

### Step 3: Decision Framework for Best Evidence Dosing Guidelines

In order to aid the possible implementation of our model-informed doses as best-evidence dosing guidelines of our simulations, we determined the following framework to evaluate the models. The following questions were aimed to evaluate the level of uncertainty of the model-informed doses and determine the doses with the best benefit-risk ratio for the intended population:

(1) What is the level of certainty of the target concentrations?
(2) What is the clinical risk of over- or underdosing?
(3) What is the level of certainty of the model output?
(4) Does the currently advised DPF dose result in adequate (simulated) target exposure?
(5) Which dose results in better target exposure, is this a significant improvement?
(6) Is the proposed dose practical?
(7) Is the population in the published PK model comparable to the simulated population (e.g., with respect to demographics, severity of illness, underlying disease)? If not, will this impact the dosing requirements?
(8) Overall conclusion

## Results

### Literature and Selection of Drugs

Piperacillin and amikacin were selected as the best drug candidates for this study, as piperacillin has three available pop-PK models, all providing a dose advice for critically ill children (Nichols et al., 2015; De Cock et al., 2017; Béranger et al., 2018) and one published amikacin pop-PK model was available, but this study did not provide a dose advice for critically ill children (Sherwin et al., 2014).

Pop-PK models were available for *piperacillin* from studies by Béranger et al., De Cock et al. and Nichols et al., which included 67, 47, and 12 PICU patients, respectively (Nichols et al., 2015; De Cock et al., 2017; Béranger et al., 2018). Age of patients ranged from 1 m to 18 y, and patients with renal dysfunction were either excluded beforehand or not included in the final study. Béranger and Nichols identified piperacillin PK was best fitted by a one compartment model, De Cock developed a 2-compartmental model.

The 2-compartment amikacin model by Sherwin et al. included 232 amikacin concentrations from 70 critically ill, pediatric burn patients, with ages ranging from 6 m up to 17 y (17). An overview of model and patient characteristics is shown in Table 1.

**TABLE 1:** Overview of study characteristics, populations, PK parameters and dose advice in the used pop-PK models.

Author	Drug	Dose regimen used in study	Population	Median age + weight (range)	Covariates final model	PK parameters	Dose advice
Béranger	PIP	300 mg/kg/d, 4 daily doses, 30 min infusion	67 critically ill children	2.3–2.6 y (1–18 y)	Cl: weight, eGFR	PIP Cl 0.18 L/kg/h	400 mg/kg/d CON or EXT
				11.9–13.7 kg (2.7–53 kg)	Vd: PELOD-2	PIP Vd 0.351 l/kg
De Cock	PIP	300 mg/kg/d, 4 daily doses, 5–30 min infusion	47 critically ill children	2.83 y (2 m–15 y)	Cl: weight, PMA	PIP Cl 0.25 L/kg/h	75 mg/kg loading dose +400 mg/kg/d CON
				14 kg (3.4–45 kg)	Vd: weight	PIP central Vd 0.13 L/kg, peripheral Vd 0.11 L/kg	
Nichols	PIP	300 mg/kg/d, 3 daily doses, 3 h infusion	12 critically ill children	5 y (1–9 y)	Cl: weight	PIP Cl 0.199 L/kg/h	100 mg/kg every 6–8 h as EXT
				18.3 kg (9.5–30.1 kg)	Vd: -	PIP Vd 0.366 L/kg	
Sherwin	AMI	10–20 mg/kg/d, 2–4 daily doses, 30 min infusion	70 critically ill pediatric burn patients	4.5 y (0.5–17 y)	Cl: weight	AMI Cl 0.085 L/h/kg	No dose advice
				20 kg (8–90 kg)	Vd: weight	AMI central Vd 0.239 L/kg, peripheral Vd 0.573 L/kg	

AMI, amikacin; Cl, clearance; CON, continuous infusion; eGFR, estimated glomerular filtration rate; EXT, extended infusion; PELOD-2, Pediatric Logistic Organ Dysfunction two score; PIP, piperacillin; PMA, postmenstrual age; Vd, volume of distribution.

### Simulation Patient Population

After exclusion of duplicate entries and patients with missing demographic data, the patient dataset included 307 patients in total, with a median age of 4.9 y (Table 2). Creatinine concentrations were available for 77 patients, with a median eGFR of 115.2 ml/min/1.73 m^2^. Disease severity scores (Pediatric Logistic Organ Dysfunction (PELOD)-2 scores), which were a covariate in the Béranger piperacillin model, could not be obtained from our hospital records, so the mean population value from the Béranger study (PELOD-2 = 4) was used (Béranger et al., 2018).

**TABLE 2:** Demographic and clinical characteristics of the Radboudumc PICU-dataset from 2018 (n = 307).

Demographic variables	Median (IQR) [range] or n (%)
Gender	
Male	164 (53.4%)
Female	143 (46.6%)
Postnatal age	4.9 y (1.2–11.5) [0.1–17.9]
Age categories	
1 m–1 y	66 (21.5%)
1–2 y	40 (13.0%)
2–4 y	30 (9.8%)
4–8 y	59 (19.2%)
8–12 y	39 (12.7%)
12–18 y	73 (23.8%)
Weight	18.0 kg (10.0–38.0) [2.1–98.0]
eGFR (n = 77)	115.2 ml/min/1.73 m^2^ (93.5–143.1) [20.9–196.2]

PICU, pediatric intensive care unit; eGFR, estimated glomerular filtration rate.

### Concentration (PK) Targets for Selected Drugs

The PK target associated with piperacillin efficacy is the percentage of time the unbound plasma concentration exceeded the minimal inhibitory concentration (%fT/MIC), which should be 100% based on latest consensus (Nichols et al., 2015; De Cock et al., 2017; Béranger et al., 2018). For our simulations we used a target concentration of 16 mg/L, which is the clinical breakpoint of *Pseudomonas aeruginosa*, as a worst-case scenario [European Committee on Antimicrobial Susceptibility Testing (EUCAST)]. The fraction of unbound piperacillin was assumed to be 70% (Piperacillin - Summary of Product Characteristics), similar to the assumed level of protein binding in the models (Nichols et al., 2015; De Cock et al., 2017; Béranger et al., 2018). The PTA for reaching the piperacillin PK-target (unbound Cmin > 16 mg/L) was assessed for the advised dosing regimens by Béranger, de Cock, Nichols and the DPF dose across the three models. Overall PTA was defined as the mean PTA across all models.

For amikacin a Cmax/MIC-ratio of 8–10 is the most commonly defined PK target for efficacy and associated with optimal bacterial killing (Tsai et al., 2015). Additionally, the safety target for amikacin is a Cmin < 5 mg/L, which is associated with a reduced risk of ototoxicity and nephrotoxicity (Zorginstituut Nederland. Amikacine. Farmacotherapeutisch Kompas, Roberts et al., 2012). Sherwin et al. used target Cmax and Cmin of 25–30 mg/L and 4–8 mg/L, respectively (Sherwin et al., 2014). As the epidemiological cut-off value obtained from EUCAST for most bacteria is 8 mg/L [European Committee on Antimicrobial Susceptibility Testing (EUCAST)] we used a target Cmax of 60–80 mg/L to ensure the target Cmax/MIC ratio to be at least 8, a target also used in critically ill adults with severe infections (de Montmollin et al., 2014). Unbound amikacin concentrations were not taken into account, as amikacin protein binding is negligible (Amikacin Summary of Product Characteristics SmPC, 2020).

### Dose Simulations - Piperacillin

As input for the simulations we used the dosing advice provided in the articles: Béranger et al. (Béranger et al., 2018) advised 400 mg/kg/d as a continuous infusion, De Cock et al. (De Cock et al., 2017) advised a loading dose of 75 mg/kg followed by a 400 mg/kg/d continuous infusion, and Nichols et al. (Nichols et al., 2015) advised 400 mg/kg/d as extended 3-h intermittent infusions every 6 h.

**FIGURE 1:**
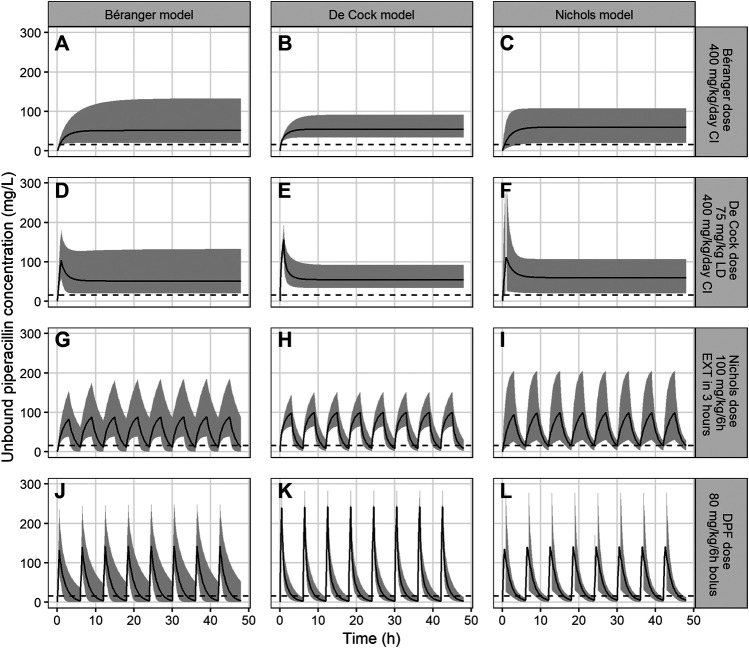
Unbound piperacillin concentrations over 48 h for models of Béranger, De Cock and Nichols. The black line represents the median piperacillin concentration, the shaded grey area the 95% prediction interval, and the dotted line the target Cmin of >16 mg/L. Columns represent the simulations of a single model (panel **(A)**, **(D)**, **(G)** and **(J)** for the Béranger model, panel **(B)**, **(E)**, **(H)** and **(K)** for the De Cock model and panel **(C)**, **(F)**, **(I)** and **(L)** for Nichols model). The rows represent different dosing recommendations from the different models (panel **(A)**, **(B)** and **(C)** for the dose proposed by Béranger (400 mg/kg/d as continuous infusion), panel **(D)**, **(E)** and **(F)** for the dose proposed by De Cock (75 mg/kg loading dose +400 mg/kg/d as continuous infusion), panels **(G)**, **(H)** and **(I)** for the dose proposed by Nichols (100 mg/kg/6 h as extended infusion during 3 h) and panels **(J)**, **(K)** and **(L)** for the current DPF dose (80 mg/kg/6 h as bolus infusion)).

Simulated concentration-time profiles for piperacillin were compared to concentration-time plots in the original publications. Our simulations showed reasonable representation of median Cmax, Cmin and interindividual variability in the original studies. Concentration-time profiles of these simulations and cross-checks between different models and piperacillin dosing regimens, are presented in Figure 1.

Both continuous dosing recommendations, by Béranger and De Cock, resulted in the highest Cmin concentrations. For the dosing regimen of Béranger steady-state median (95% prediction interval) piperacillin concentrations were 51.2 mg/L (20.0–134.0), 54.8 mg/L (33.8–91.1) and 59.8 mg/L (20.3–107.2) in the models of Béranger, De Cock and Nichols, respectively (Figure 1, first row). The dose regimen proposed by De Cock et al. (400 mg/kg/d as continuous infusion with a 75 mg/kg loading dose) yielded similar median concentrations 51.4 mg/L (19.7–132.6), 54.8 mg/L (33.5–90.5) and 59.8 mg/L (20.2–107.8) (Figure 1, second row), but reached therapeutic concentrations faster. Contrarily, regimens using intermittent doses, as advised by Nichols et al. and the DPF dose, did not reach median Cmin > 16 mg/L (Figure 1, third and fourth row).

**FIGURE 2:**
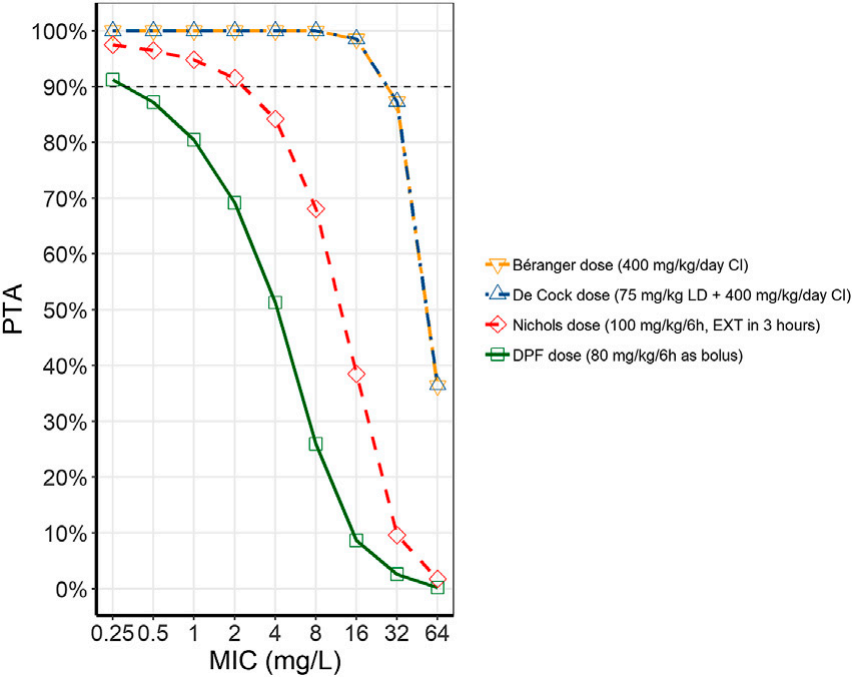
Probability of target attainment (PTA) for piperacillin with different dosing regimens. The different lines represent the average PTA across the three models at different MICs for the different dosing regimens (Béranger dose = yellow, dashed line, downward facing triangle; De Cock dose = blue, dash-dotted line, upward facing triangle; Nichols dose = red, dashed line, diamonds; DPF dose = green, solid line, squares). (CI, continuous infusion; DPF, Dutch Pediatric Formulary; EXT, extended infusion; LD, loading dose; MIC, minimum inhibitory concentrations).

PTA of piperacillin at a MIC of 16 mg/L was > 90% in for both continuous dosing regimens by Béranger and de Cock (Figure 2) across all three models. The intermittent dosing regimen of Nichols (400 mg/kg/d, 6 h dosing interval, extended infusion of 3 h) showed an overall PTA of 36.7%, ranging from 19.2% in the de Cock model to 47.7% in the Nichols model. However, this is still markedly higher than what is reached with the current DPF dosing regimen, with an overall PTA of 9.6%, ranging from 0.6–25.8% in the Nichols and Béranger model respectively.

### Dose Simulations - Amikacin

**FIGURE 3:**
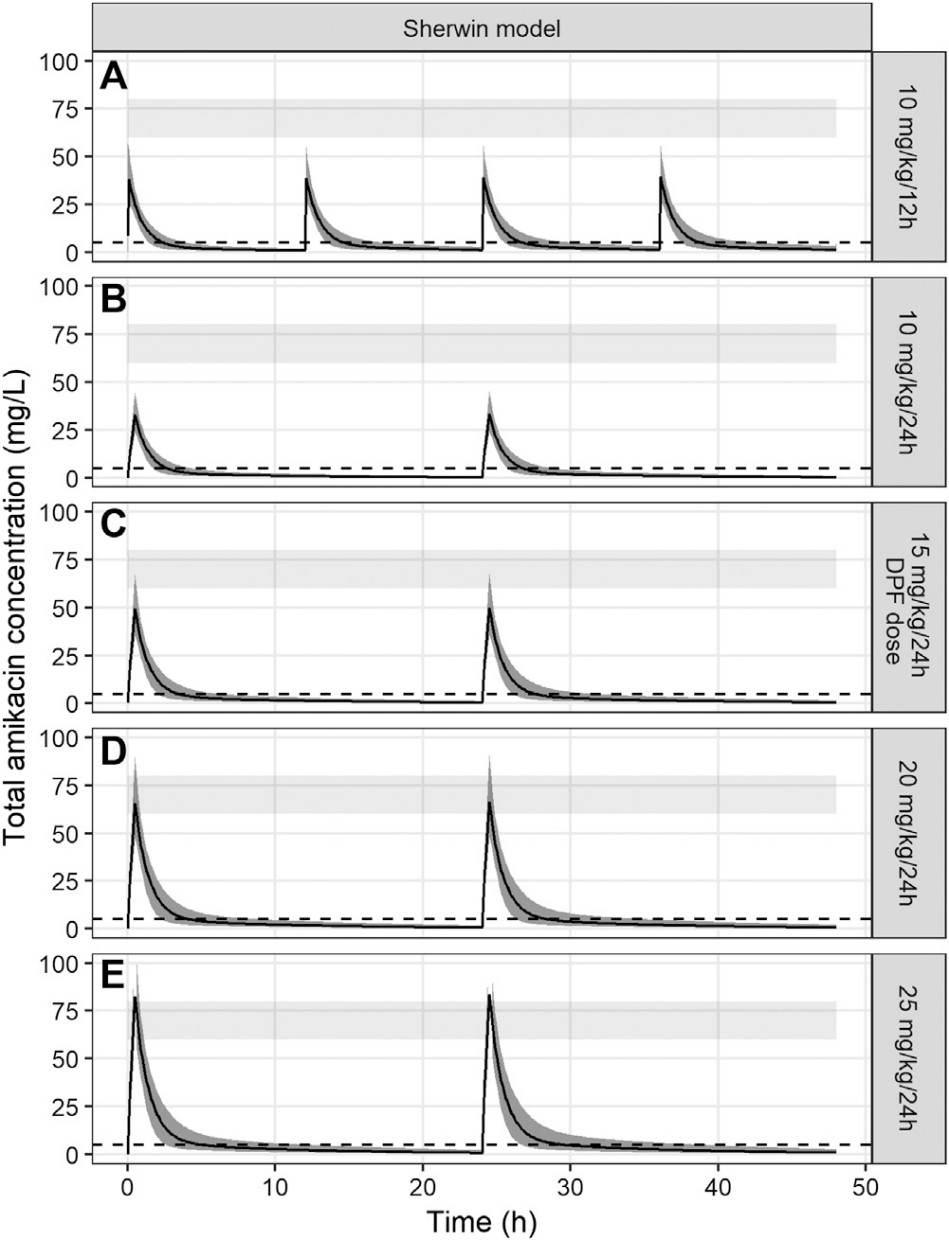
Amikacin concentration-time curves simulated over 48 h using the PK model of Sherwin et al. The black line represents the median amikacin concentration, the dark grey area around the line the 95% prediction interval. The light grey band represents the target Cmax (60–80 mg/L) and the dotted line represents the target Cmin (<5 mg/L). Panels **(A)**–**(E)** represent different tested dosing regimens, including the currently advised DPF dose (panel **(C)**).

There was no dosing advice presented in the Sherwin article, so the starting point for the simulations was the dosing advice in the DPF (15 mg/kg every 24 h) and the highest registered dose (20 mg/kg/d) in the summary of product characteristics (SmPC) (Amikacin Summary of Product Characteristics SmPC). Subsequently, a trial and error-method resulted in a concentration course over time, for which the predetermined PK-targets for effectivity and toxicity were reached (Figure 3).

Dosing regimens of 10 mg/kg/24 h, 15 mg/kg/24 h, 10 mg/kg/12 h, 20 mg/kg/24 h and 25 mg/kg/24 h were tested. The dosing regimen of 20 mg/kg every 24 h, administered over 30 min, reached predetermined PK-target in most patients, with a simulated Cmax of 70.2 mg/L (95% prediction interval 51.7–97.5) and for Cmin 1.1 mg/L (95% prediction interval 0.3–2.3). Other dosing regimens demonstrated suboptimal results: all dosing regimens under 20 mg/kg/dose failed to reach appropriate Cmax concentrations (33.9 mg/L (24.6–45.0) for 10 mg/kg/dose and 50.6 mg/L (35.5–68.3) for 15 mg/kg/dose). On the other hand, a regimen of 25 mg/kg/24 h resulted in supratherapeutic Cmax concentrations (82.3 mg/L (55.9–107.5)). PTA of the safety target (Cmin < 5 mg/L) was 100% for all simulated dosing regimens.

**FIGURE 4:**
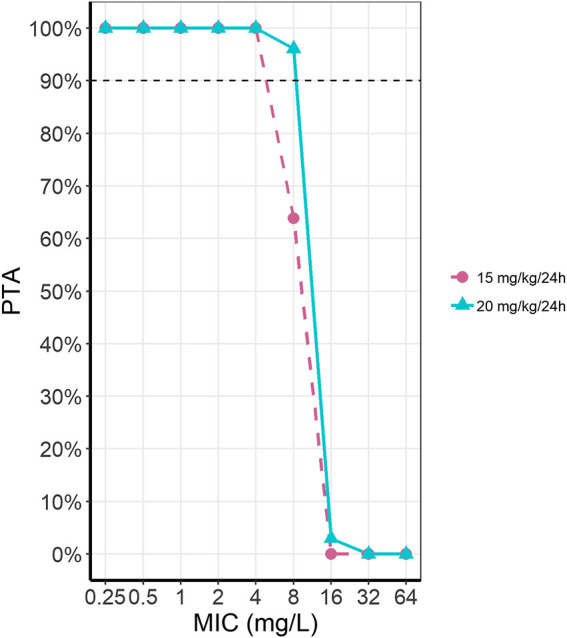
Probability of target attainment (PTA) for amikacin for two dosing regimens at different MICs. The two presented dosing regimens are the current DPF recommendation of 15 mg/kg/24 h (pink, dashed line, circles) and our optimally simulated dose of 20 mg/kg/24 h (light blue, solid line, triangles).

Probability of target attainment (PTA) was simulated for the currently proposed dosing regimen from the DPF (15 mg/kg/24 h) and our proposed dose of 20 mg/kg/24 h (Figure 4). For an MIC of 8 mg/L, the current dose reaches a PTA of 63.5%, while a dosing regimen of 20 mg/kg/24 h reaches a PTA of 96.2%. Differences in PTA between these two dosing regimens for other MICs was minimal.

### Decision Framework for Best Evidence Dosing Guidelines

#### Piperacillin

(1) What is the level of certainty on the target concentrations?
  Moderate to high, based on EUCAST MIC concentrations and widely accepted definition of target attainment %fT/MIC >100. The percentage unbound drug is estimated, in line with other studies of this drug in this population.
(2) What is the clinical risk of over- or underdosing?
  In general penicillins show a relatively mild safety profile. The additional risk of overdosing of tazobactam, the accompanying drug in all piperacillin formulations, should be taken into account. However, this also appears to be relatively safe in higher than licensed doses (McDonald et al., 2016). Underdosing may result in ineffective bacterial clearance, which weighs heavier than the relatively mild side-effects, especially in critically ill patients.
(3) What is the level of certainty of the model output?
  Simulation of concentrations using all three models resulted in similar exposures as in the publications. Moreover, all studies used state of the art internal validation methods. However, interindividual and residual variability was relatively large in all three models, which widens the prediction intervals of our simulations.
(4) Does the currently advised DPF dose result in adequate target exposure?
  No, only 9.6% reaches the target Cmin concentration of >16 mg/L with the current intermittent dosing regimen of 320 mg/kg/d as intermittent dose.
(5) Which dose results in better target exposure, is this a significant improvement?
  400 mg/kg/d performed significantly better than the DPF daily dose of 320 mg/kg/d. Continuous infusion performed best (PTA > 90%), and when combined with a loading dose of 75 mg/kg this is the most optimal dosing regimen to reach fast and steady therapeutic concentrations.
(6) Is the proposed dose practical?
  Continuous infusions may not be practical in critically ill children where venous access is always challenging and limited. Intermittent doses using an extended dosing interval may be more practical in clinical practice.
(7) Is the population in the published PK model comparable to the simulated population (e.g., with respect to demographics, severity of illness, underlying disease)? If not, will this impact the dosing requirements?
  The models all included critically ill children with mixed underlying reasons for ICU admission which did cover most of the pediatric age range, but with a slightly lower median age compared to our simulation cohort. Therefore, these results might be applicable to critically ill children, but less applicable for non-critically ill children, although piperacillin-tazobactam will likely only be prescribed to severely ill children. Additionally, we used a worst-case scenario for MIC, whereas actual MIC targets may differ for other bacteria or other areas where microbial resistance may be different (Woksepp et al., 2016).
(8) Overall conclusion for piperacillin:
  According to our simulations, the proposed optimal dosing regimen for critically ill children is a loading dose followed continuous infusion to reliably reach target concentrations shortly after diagnosis (De Cock et al., 2017). In situations where continuous infusion is not possible, the alternative option would be the Nichols dosing regimen of 400 mg/kg/d as an extended 3-h infusion (Nichols et al., 2015). For non-critically ill children, the current dosing advice could be continued, although a similar simulation study for non-critically ill children also suggested a slightly higher dose of 360 mg/kg/d and extended infusion in 2 h to reach adequate targets (Thibault et al., 2017). Additionally, as dose-related toxicity is limited, harmonizing the dose across the pediatric populations would be more practical.

#### Amikacin

(1) What is the level of certainty on the target concentrations?
  Moderate, based on EUCAST MIC concentrations and a common definition of target attainment: Cmax/MIC ratio to be at least 8, a target also used in critically ill adults with severe infections. However, others use less aggressive target Cmax, possible in a setting with less microbial resistance. Additionally, although Cmax/MIC is the most commonly used target, a recent publication also proposes AUC/MIC to be the optimal target for aminoglycoside efficacy (Bland et al., 2018).
(2) What is the clinical risk of over- or underdosing?
  Amikacin’s dose-related toxicity is kidney failure and ototoxicity related to the Cmin. A meta-analysis of amikacin side-effects in adults shows a prevalence of nephrotoxicity and irreversible ototoxicity of 5.3% and 8.6%, respectively (Jenkins et al., 2016), which may cause a major burden on healthcare and patient lives. Underdosing may result in ineffective bacterial clearance, and potentially life-threatening infections and/or sepsis. Therefore, therapeutic drug monitoring (TDM) is routinely advised for aminoglycosides, so the dosing regimens of an individual patient can be adjusted to ensure therapeutic, non-toxic amikacin exposure.
(3) What is the level of certainty of the model output?
  Moderate. Simulations of concentrations using the model parameters resulted in similar exposures as published. Moreover, the study used state of the art internal validation methods, but external validation is missing.
(4) Does the currently advised DPF dose result in adequate target exposure?
  No, the DPF dose results in a PTA of 63.5% at MICs of 8 mg/L.
(5) Which dose results in better target exposure, is this a significant improvement?
  20 mg/kg/d results in better PTA at the same MIC (96.2%), with similar (non-toxic) Cmin. For patients infected with a micro-organism of this MIC this would be a significant improvement. However, target attainment for different MICs is comparable between 15 and 20 mg/kg/d.
(6) Is the proposed dose practical?
  Yes.
(7) Is the population in the published PK model comparable to the simulated population (e.g., with respect to demographics, severity of illness, underlying disease)? If not, will this impact the dosing requirements?
  The model population consisted of a highly specific subgroup of critically ill children, pediatric burn patients (Sherwin et al., 2014). It is known that due to e.g., fluid retention, pharmacokinetics may differ in patients with extensive burn injury, resulting in lower exposures. Hence, the data cannot be automatically extrapolated to non-burned critically ill children. The study does cover the full pediatric age range within their cohort.
(8) Overall conclusion for amikacin:
  Although the simulated dosing regimens suggested a higher daily dose (20 mg/kg/d) than the current DPF, the difference in patient population, potentially explaining the difference with the current DPF dose, the risk of dose-related toxicity in another (critically ill) population, and the relatively small benefit limited to MICs of 8 mg/L does not support an overall change in DPF dose. In critically ill pediatric burn patients, this higher dose could be considered, but only in the absence of renal failure and with strict TDM.

## Discussion

In this proof of concept study we explored the feasibility to develop a framework to aid the implementation of model-based dosing guidelines using published pop-PK models in critically ill children, with piperacillin and amikacin as examples. We found that this model-based strategy is feasible to use in practice and we were able to compare dosing advices from three different models of piperacillin, which showed marked differences in PTA between doses advised in the studies. Additionally, we generated a simulated dose for amikacin, an antibiotic for which the dosing advice was not provided in the paper describing the model (Sherwin et al., 2014). Lastly, we used a standardized framework of questions to explore whether these findings warrant a change in the dosing regimen advised by the DPF or other pediatric drug handbooks.

Simulations for both antibiotics suggest that the current DPF dosing regimen results in a suboptimal target attainment in critically ill children. For piperacillin, the evidence is more apparent, as all three articles propose at least 400 mg/kg/d for adequate exposure specifically for critically ill children (Nichols et al., 2015; De Cock et al., 2017; Béranger et al., 2018). These results might warrant an alteration in the DPF dosing recommendation, as supported by our decision framework, which also takes study quality and clinical benefit and risks into account.

For amikacin, while the simulation suggests a higher daily dose, our decision framework does not support a change in dosing regimen. Our simulation results may be only applicable for critically ill children with severe burn injury, a highly specific subgroup with unique pharmacokinetic challenges (Sherwin et al., 2014). Severe burn injury induces several pathophysiological alterations, including capillary leak, extreme interstitial edema, hypovolemia and reduced organ perfusion in the early phase (Udy et al., 2018). Furthermore, treatment of severe burn patients revolves around large volumes of IV fluid resuscitation to increase intravascular pressure and organ perfusion, but also leading to additional extracellular fluid accumulation (Udy et al., 2018). Both pathophysiological alterations and therapies contribute to markedly higher Vd of hydrophilic drugs, like amikacin (Tsai et al., 2015). Additionally, the second phase of burn injury typically involves organ hyperperfusion, which may cause augmented renal clearance making critically ill, burn patients a highly challenging subgroup to dose correctly (Udy et al., 2014; Udy et al., 2018).

In the Sherwin cohort these pharmacokinetic changes are evident, as total amikacin Vd was markedly higher (0.81 L/kg) compared to non-burned infants (0.337 L/kg) (Treluyer et al., 2002). The most commonly used PK-target for amikacin efficacy (Cmax/MIC), is largely influenced by this larger Vd resulting in a higher dose to reach similar Cmax. Additionally, aminoglycosides concentrations are subject to routine TDM, so potential reduced exposure with the current regimen can corrected when necessary. While simultaneously, a higher dose may result in irreversible toxicity in non-burn patients. Therefore, a nationwide dose alteration might not be warranted at the moment.

Pharmacokinetic studies of other antibiotic agents in critically ill children, also suggest similar reduced (simulated) target attainment (Jenkins et al., 2016; McDonald et al., 2016; Bland et al., 2018). Unfortunately, a direct and practical translation to clinical practice is lacking. Not only are model-informed doses frequently not proposed in these manuscripts, even if they are, authors are very reluctant to support clinical implementation as they consider further validation unnecessary. We do support high quality data to establish with large certainty the correctness of model-informed doses (Ince et al., 2009). At the same time, in the absence of more data and the practice of off-label prescribing, not using existing pharmacokinetic data to add to the current evidence-base that supports doses used in real-life clinical care is a missed opportunity. Our decision framework helps to interpret study results with the aim to translate findings to the clinical setting, in a similar risk-benefit analysis that is currently applied by the DPF (van der Zanden et al., 2017). This allows for a thorough evaluation of study results not only for pharmacological efficacy, but also for toxicity, practicality and assessment of external validity of research findings to another clinical setting.

Although our model-based approach to generate evidence-based dosing recommendations seems feasible, it comes with some limitations. The quality of the model-based results is largely influenced by the quality and population of the data provided in literature. We used only pop-PK model data to ensure the highest possible quality of PK parameter estimates, but the models were not externally validated and for amikacin the only available PICU study was performed in burn patients which limits the external validity of our findings. Ideally, future research towards this approach should also include lower-quality data, for example non-compartmental estimates of Vd and Cl, but accurate simulations of drug exposure might prove difficult with suboptimal data. Secondly, this method benefits from established target concentrations that correlate with either effect, drug toxicity or both, which is not available for all drug classes. Thirdly, we have generated a dosing advice using a trial-and-error approach and with a relatively small virtual PICU cohort, which might be further optimized by more advanced modelling techniques and a larger database of virtual patients including more covariate data. Lastly, although we aimed to close the knowledge gap for evidence-based dosing in children with limited resources, it is of the essence to not abandon the drug dosing quality improvement cycle and evaluate the updated dosing regimens (Ince et al., 2009).

Ideally all dosing recommendations would be based on the most robust form of pharmacokinetic evidence, e.g., from large clinical PK trials or robust pop-PK studies. Additionally, routine TDM strategies could be applied in clinical practice to ensure therapeutic and non-toxic drug exposure after the starting dose has been given. Given the large inter-individual variability in critically ill children, the relatively well-known PK targets associated with efficacy and/or safety, and the inability to judge therapeutic effect by other parameters, routine TDM for antibiotics could be beneficial in this patient population (Wallenburg et al., 2020). Ultimately, model-informed precision dosing applications that are currently under development can translate these robust PK data into tailored dosing regimens for an individual patient, using TDM samples and Bayesian feedback to further improve and individualize dosing of antibiotics in special populations (Darwich et al., 2017; Keizer et al., 2018).

However, reality shows overall dosing recommendations for children regularly need to be made using suboptimal, best-evidence data. In this dilemma between costly, high-grade, patient-specific evidence and pragmatic, best-evidence dosing guidelines, our method could be used as an additional tool to improve current dosing guideline practices. This method is relatively easy to apply by professionals involved in pediatric dosing guidelines, even without prior modelling experience, as it only requires basic knowledge on model structures and R. Within current practice, it can serve to compare multiple models and can be used to simulate dosing regimens using real PK data. Future studies regarding this method should focus on the applicability in a clinical setting, like the DPF. If not implemented properly, this might harm reproducibility and ease of use in practice.

## Conclusion

Our framework using existing PK data to established model-informed doses can be used as a relatively affordable, easy and efficient simulation tool in special populations, and can be used in conjunction with current strategies for developing evidence-based dosing recommendations. We have shown for piperacillin in critically ill children a higher dose might be warranted. In contrast, for amikacin population differences, uncertainty on target exposure, increased risk of toxicity and small benefits, do not support a change in the clinical dosing guidelines. Although this method cannot replace well-designed clinical trials, it can prove to be valuable, especially for the pediatric population, where off-label prescribing remains very prevalent.

## Data Availability Statement

The raw data supporting the conclusion of this article will be made available by the authors, without undue reservation.

## Author Contributions

SH and SW published systematic review that served as a basis for current work. JS, SH, BG, and SB carried out practical work of current study (data cleaning, building simulation script and performing simulations). RH, BG, TZ, MH, and SW provided supervision support over the course of the current study. JS and SH wrote the first version of the manuscript. Final version of the manuscript written by SH, JS, SB, BG, TZ, MH, RH, and SW. Overall, SH and JS have contributed equally.

## Conflict of Interest

SW is Medical Director of the Dutch Pediatric Knowledge Center Pharmacotherapy for Children and as such responsible for the Dutch Pediatric Formularies and its international editions. TZ is managing director of the Dutch Paediatric Pharmacotherapy Expertise Network and as such responsible for the Dutch Pediatric Formularies and its international editions. MH is pharmacist for the Dutch Pediatric Formulary.

The remaining authors declare that the research was conducted in the absence of any commercial or financial relationships that could be construed as a potential conflict of interest.
